# Simultaneous Blockade of Histamine H_3_ Receptors and Inhibition of Acetylcholine Esterase Alleviate Autistic-Like Behaviors in BTBR T+ tf/J Mouse Model of Autism

**DOI:** 10.3390/biom10091251

**Published:** 2020-08-28

**Authors:** Nermin Eissa, Petrilla Jayaprakash, Holger Stark, Dorota Łażewska, Katarzyna Kieć-Kononowicz, Bassem Sadek

**Affiliations:** 1 Department of Pharmacology & Therapeutics, College of Medicine and Health Sciences, United Arab Emirates University, Al Ain P.O. Box 17666, UAE; nermineissa@uaeu.ac.ae (N.E.); petrilla.jp@uaeu.ac.ae (P.J.); 2 Zayed Center for Health Sciences, United Arab Emirates University, Al Ain P.O. Box 17666, Abu Dhabi, UAE; 3 Department of Applied Sciences, College of Arts and Sciences, Abu Dhabi University, Al Ain Campus, Al Ain P.O. Box 59911, Abu Dhabi, UAE; 4 Institute of Pharmaceutical and Medicinal Chemistry, Heinrich Heine University Düsseldorf, Universitaetsstr. 1, 40225 Düsseldorf, Germany; stark@hhu.de; 5 Department of Technology and Biotechnology of Drugs, Faculty of Pharmacy, Jagiellonian University-Medical College, Medyczna 9 St., 30-688 Kraków, Poland; dlazewska@cm-uj.krakow.pl (D.L.); mfkonono@cyf-kr.edu.pl (K.K.-K.)

**Keywords:** autism spectrum disorder, BTBR mice, histamine, histamine H_3_ receptor antagonist, acetylcholine, acetylcholine esterase inhibitor, cerebellum, ASD-like features, microglial cells

## Abstract

Autism spectrum disorder (ASD) is a heterogenous neurodevelopmental disorder defined by persistent deficits in social interaction and the presence of patterns of repetitive and restricted behaviors. The central neurotransmitters histamine (HA) and acetylcholine (ACh) play pleiotropic roles in physiological brain functions that include the maintenance of wakefulness, depression, schizophrenia, epilepsy, anxiety and narcolepsy, all of which are found to be comorbid with ASD. Therefore, the palliative effects of subchronic systemic treatment using the multiple-active test compound E100 with high H_3_R antagonist affinity and AChE inhibitory effect on ASD-like behaviors in male BTBR T+tf/J (BTBR) mice as an idiopathic ASD model were assessed. E100 (5, 10 and 15 mg/kg, i.p.) dose-dependently palliated social deficits of BTBR mice and significantly alleviated the repetitive/compulsive behaviors of tested animals. Moreover, E100 modulated disturbed anxiety levels, but failed to modulate hyperactivity parameters, whereas the reference AChE inhibitor donepezil (DOZ, one milligram per kilogram) significantly obliterated the increased hyperactivity measures of tested mice. Furthermore, E100 mitigated the increased levels of AChE activity in BTBR mice with observed effects comparable to that of DOZ and significantly reduced the number of activated microglial cells compared to the saline-treated BTBR mice. In addition, the E100-provided effects on ASD-like parameters, AChE activity, and activated microglial cells were entirely reversed by co-administration of the H_3_R agonist (*R*)-α-methylhistamine (RAM). These initial overall results observed in an idiopathic ASD mice model show that E100 (5 mg/kg) alleviated the assessed behavioral deficits and demonstrate that simultaneous targeting of brain histaminergic and cholinergic neurotransmissions is crucial for palliation of ASD-like features, albeit further in vivo assessments on its effects on brain levels of ACh as well as HA are still needed.

## 1. Introduction

Autism spectrum disorder (ASD) is a construct used to describe individuals with a specific combination of core behavioral persistent deficits in social interactions and the presence of patterns of repetitive and restricted behaviors [1]. The pathophysiology of ASD remains poorly understood, regardless of its worldwide increasing prevalence [2,3]. The complexity in understanding the pathophysiology of ASD lies in the heterogeneity of the disorder and its co-occurrence with other psychiatric or neurological disorder, making diagnosis and clinically specific targeted interventions for ASD often less efficient [4,5].

Studies focused on modeling ASD in rodents based on ASD-related behaviors clinically observed in individuals with ASD, such as stereotyped and repetitive behaviors and impairments in social interaction and communication. In this regard, the inbred BTBR T+ tf/J (BTBR) mice has been used as an animal model that garnered attention related to ASD because the BTBR mice exhibits behaviors consistent with the diagnostic categories for ASD, namely social impairment and obsessive/compulsive behaviors, e.g., increased self-grooming, marble burying and disturbed anxiety levels [6,7,8].

In patients diagnosed with ASD, significant abnormalities in the brain cholinergic system particularly in the structure and number of neurons in the basal forebrain cholinergic nucleus have been reported [9]. In addition, a significant choline deficiency, a precursor molecule for the synthesis of acetylcholine (ACh) neurotransmitter and nicotinic-cholinergic receptor (nAChR) agonist, was reported in brains of persons with ASD [10]. Also, alterations in the levels of nAChRs were observed in numerous areas of the brain including neocortex, thalamus, striatum and cerebellum of ASD individuals, with the central abnormality being the diminishment of muscarinic receptors (M1 subtype) [10,11].

The brain histaminergic system controls several essential physiological and higher brain functions e.g., wakefulness and attention, sensory and motor functions, energy and endocrine homeostasis, cognition and memory and as such, are all severely affected in neuropsychiatric disorders including ASD [5,12,13,14,15]. Brain histamine (HA) provides its effects through binding to designated histamine H_1_ to H_4_ receptors (H_1_R–H_4_R) belonging to the G-protein-coupled receptors family. Initially in 1983 it was found that histamine H_3_ receptor (H_3_R) negatively regulate HA synthesis and release with high constitutive activity, acting presynaptically as auto-receptors [16,17]. In addition, the functioning of H_3_ hetero-receptors (H_3_Rs) modulate the release of different neurotransmitters (e.g., ACh, serotonin (5-HT), dopamine (DA), γ-aminobutyric acid (GABA), noradrenaline (NA) and glutamate (Glu) in various regions of the brain [18,19,20,21,22]. Moreover and in the central nervous system (CNS), H_3_Rs are chiefly expressed on the histaminergic neurons.

Interestingly, limited studies proposed the potential clinical use of H_3_R antagonists in the therapeutic management of autistic behavior. Consequently, the imidazole-based H_3_R ligand ciproxifan [23] and the non-imidazole-based H_3_R antagonist DL77 were described to demonstrate promising enhancing effects on valproic acid-induced ASD-like features in mice [24]. Moreover, we recently showed that the dual-active H_3_R antagonist and acetylcholine esterase (AChE) inhibitor E100 mitigated oxidative stress and proinflammatory cytokines and alleviated social deficits and stereotyped repetitive behaviors in C57BL/6 mice exposed to valproic acid [8,25]. Furthermore, it has been proposed that histamine H_3_R antagonists/inverse agonists are of potential therapeutic future application for the treatment of CNS-related disorders, such as, Alzheimer’s disease (AD), depression, schizophrenia (SCH) and narcolepsy [26,27,28]. Accordingly, H_3_R antagonist was found to revert behavioral impairments in animal model of SCH (considered as a neuropsychiatric disorder with spatial working memory deficit) with several behavioral features that are also observed in individuals with ASD [29]. Moreover, antagonism of H_3_Rs was found to mitigate deficits in social behaviors by rodents exposed to phencyclidine, signifying the potential efficacy of H_3_R antagonists that may be promising in the therapeutic management of ASD [30].

Several preclinical experimental observations revealed that cerebellar inflammation may affect social behaviors in adult mice, since cerebellum was found to be implicated in executive behavioral functions and cognition [31,32,33,34]. Interestingly collective neuropathological studies on autism post-mortem brains has identified that cerebellum contributes as one of the key brain regions closely linked to many if not most of the behavioral symptoms observed in ASD [35] and substantial accumulating evidence has associated the cerebellum with higher cognitive functions [36]. Moreover, the cerebellum is being considered a key structure within the social circuitry highlighting the crucial role for the cerebellum in the etiology of ASD [37].

To comprehend our previous results observed for the dual-active compound E100 in valproic acid-induced ASD in mice and as a continuation of our research, in the present study we describe the in vivo effects of E100 (1-(7-(4-chlorophenoxy) heptyl)azepine) with balanced AChE inhibitory effect (*Ee*AChE: IC_50_ = 2 µM and *Eq*BuChE: IC_50_ = 2 µM). E100 is characterized as H_3_R antagonist with affinity (*h*H_3_R *K*_i_ = 203 nM) and high selectivity profile towards H_3_R subtype and was assessed on deficits in social features and the presence of repetitive and restricted behaviors in BTBR mice as an idiopathic mouse model of ASD. Furthermore, E100 was tested for its effects on anxiety-like behaviors in the same animals, through the elevated plus-maze test (EPMT) and open field test (OFT). In addition, the effects of E100 on microglial cells and AChE activity were evaluated in the cerebellum, as the cerebellum is involved in numerous executive and cognitive functions that may alter social behaviors in adult mice [31,33,34]. To comprehend our results, the H_3_R agonist (*R*)-α-methylhistamine (RAM) ability to reverse the E100 provided effects were evaluated to elucidate the possible involvement of brain HA and ACh in the effects observed for E100.

## 2. Material and Methods

### 2.1. Animals

C57BL/6 (C57) and BTBR T+tf/J (BTBR) male mice aged 8–12 weeks and weighing 20–25 g (Jackson Laboratory, Bar Harbor, ME, USA) were kept in polypropylene cages (27 cm × 17 cm × 12 cm) in a controlled air-conditioned room (22°, relative humidity 30%) with a 12-h light/dark cycle (1lights on at 07:00 AM) and with free access throughout the experimental period to water and standard rodent chow diet. Both C57 (Ctrl mice) and BTBR mice began behavioral assessments at 8 weeks of age. All the procedures were carried out after the approval from the Institutional Animal Ethics Committee of United Arab Emirates University (ERA-2017-5603), in accordance with the European Communities Council recommendations Directive of 24 November 1986 (86/609/EEC).

### 2.2. Drugs and Biochemical Reagents

In the current study, all the chemical reagents including the (*R*)-α methyl histamine (RAM) the H_3_R brain penetrant agonist and donepezil hydrochloride (DOZ) were acquired from Sigma-Aldrich, St. Louis, MO, USA. The design and synthesis of E100, namely 1-(7-(4-chlorophenoxy)heptyl)azepane, was carried out in the Department of Technology and Biotechnology of Drugs, Jagiellonian University Medical College, Krakow, Poland and as described in in previous reports [38,39]. The colorimetric assay kit for AChE activity (Lot no: GR 3295454-2, Product: ab65345) was obtained from BioVision, Milpitas, CA, USA. Anti-ionized calcium binding adaptor molecule -1 (Iba-1) was purchased from Wako Chemicals, Richmond, VA, USA. Alexa Fluor^TM^ 488-conjugated goat anti-rabbit secondary antibodies were purchased from Life Technologies, Grand Island, NY, USA. All analytical grade reagents and drugs used in the study were dissolved in 1% aqueous Tween-20 solution (SAL). The volume of intraperitoneally (i.p.) administration was 10 mL/kg adjusted to body weight of mice. All doses were carefully selected based on our results from previous studies of strongly related dual-active compounds and are expressed in terms of the free bases [8,24,25,40,41,42]. E100 and DOZ or SAL i.p. treatments were administered daily for 21 days and were injected 30–45 min prior each behavioral test. A battery of behavioral assessments began one week after starting the treatments, following a regimen that has been standardized in our laboratory in concurrence with previously published protocols [8,23,24]. The behavioral experiments of the study were conducted once the animals were 50 days of age in the following sequence; three-chamber behavior (TCB) test, elevated plus maze test (EPMT), open field test (OFT), marble burying behavior (MBB) test and nestlet-shredding test (NST). The behavioral assessments were performed between 08:00 and 14:00) pm in a quiet area that was illuminated with four 60-V light-emitting diodes (LEDs). The animals were habituated in the experimental room at least for 1 h before starting the behavioral testing.

### 2.3. Behavioral Assessments

#### 2.3.1. Three-Chamber Test (TCB)

TCB evaluates social approach behaviors through social interaction and preference for social novelty, identifying sociability and/or social novelty impairments in rodents, using methods previously described [8,24,25,43]. This behavioral test has two habituation phases (center and all 3 chambers). The test mouse was habituated to the apparatus for 5 min in the center chamber only (Phase I), followed by additional 5 min with access to all 3 empty chambers (Phase II). The test mouse was then confined to the middle chamber, while a small plastic cage referred to as novel object (NO) was placed into one of the side chambers and a stranger mouse referred to as novel mouse (NM) was enclosed in an identical cup and was placed in the opposite side chamber. Stranger mice used was naïve to the test mouse and of same age and gender. The location (left or right) of the NO and NM alternated across subjects to avoid side preference. The chamber doors were then opened simultaneously, and the test mouse had to explore all 3 chambers for 10 min (Phase III). The time spent exploring the NM and NO (direct investigation) was recorded for 10 min. After the end of this session, another 10-min session (Phase IV) started providing a measure of social novelty preference. A new stranger mouse referred to as NM was added into the empty cage and the test mouse again had access to all 3 chambers for 10 min. The NM from the previous session was referred to as the familiar mouse (FM) in this test phase. EthoVision^®^ software (Noldus, version 12, Wageningen, Netherlands) automatically scored the spent exploring NO, NM and FM during each 10-min phase of the test. As described previously, the sociability index (SI) and social novelty index (SNI) were calculated to directly compare the social behaviors between treated groups [8,24,25,43]. The score for SI and SNI varied from −0.5 to +0.5; as the score became closer to 1, it indicated more sociability and social novelty preference of the animal, respectively. The followings equation was used to calculate SI and SNI:

(1) $$\mathrm{SI}\;=\;\frac{\mathrm{Time}\;\mathrm{exploring}\;\mathrm{NM}\;-\;\mathrm{Time}\;\mathrm{exploring}\;\mathrm{NO}\;}{\mathrm{Time}\;\mathrm{exploring}\;\mathrm{NM}\;+\;\mathrm{Time}\;\mathrm{exploring}\;\mathrm{NO}}$$

(2) $$\mathrm{SNI}\;=\;\frac{\mathrm{Time}\;\mathrm{exploring}\;\mathrm{NM}\;–\;\mathrm{Time}\;\mathrm{exploring}\;\mathrm{FM}\;}{\mathrm{Time}\;\mathrm{exploring}\;\mathrm{NM}\;+\;\mathrm{Time}\;\mathrm{exploring}\;\mathrm{FM}}$$

Stranger mice were habituated to the plastic cage for 30-min sessions 24 h before the test. The stranger mice were enclosed in the plastic cup in three-chamber apparatus to ensure that all social approach was initiated by the test mice. In TCB assessment, 5 mice were used per group for eight groups.

#### 2.3.2. Marble Burying Test (MBB)

MBB was performed for testing repetitive and compulsive behaviors, as described previously with slight modifications [8,24,25,42,43,44,45,46]. Briefly, empty polycarbonate cages (26 cm × 48 cm × 20 cm) were filled with 5 cm of bedding on top of which 20 glass marbles were carefully placed in the cage in 4 rows of 5 marbles each. Each mouse was allowed to explore for 30 min test session and cumulative number of marbles buried (> 50% marble covered by the bedding material) was recorded at the end of the testing session and the percentage was calculated as reported previously [24,47,48]. Each mouse was individually habituated to a marble free testing cage separately, for 10 min before the test. In MBB, 5 mice were used per group for eight groups.

#### 2.3.3. Nestlet-Shredding Test (NST)

As previously described, NST was performed by placing a mouse into a 19 × 29 × 13 cm cage with 0.5 cm bedding material on the floor, along with a preweighed commercially available cotton fiber (nestlets) [8,24,25]. Nestlets were 25 cm^2^ × 5-mm-thick cotton weighing about 2.5 g each. Mice were left undisturbed with the nestlet for 30 min after which the intact nestlet was left to dry overnight and then weighed. The percentage of nestlet shredded was calculated [49,50]. In NST assessment, 5 mice were used per group for eight groups.

#### 2.3.4. Elevated Plus Maze Test (EPMT)

The EPMT was conducted based on that described previously with slight modifications [8,24,25,49,50,51,52]. Briefly, a mouse was placed in the central zone (6 cm × 6 cm) facing 30-cm open-arm length of elevated plus maze arena constructed from Plexiglas. The maze is composed of two opposite open arms and two opposite closed arms (30 cm × 6 cm × 15 cm). Time spent and frequency of visits into each arm by each animal were recorded and analyzed for 5 min test session with EthoVision^®^ Software of version 12 (Noldus, Wageningen, Netherlands). The apparatus was carefully cleaned with 70% alcohol between subjects. In the EPMT, 5 mice were used per group for eight groups.

#### 2.3.5. Open Field Test (OFT)

OFT is a common measure of general activity, exploratory behavior and anxiety-related behavior in rodents, assessed by freely exploring a novel open-field arena (45 cm × 45 cm × 30 cm) for 5 min [8,24,25]. Animal’s activity was measured by recording the total distance traveled in the whole arena, time spent in the periphery and center (23 cm × 23 cm) for 10 min. Activity was analyzed with EthoVision (Noldus Information Technology, version 12, Wageningen, Netherlands) motion tracking apparatus and software. In OFT, 4 mice were used per group for eight groups.

### 2.4. Biochemical Assessments

#### 2.4.1. Brain Collection and Tissue Preparation for AChE Activity Assessment

Following the behavioral assessments, the animals were sacrificed according to previously published protocols [8,24,25,33]. Prior sacrifice the treated animals were deeply anesthetized with pentobarbital (40 mg/kg body weight, i.p.), followed by cardiac perfusion (0.01-M phosphate-buffered saline (PBS) at pH 7.4) to wash out the blood. The optimal pressure during the manual perfusion with 20 G needle of 50 mL syringe was obtained by slowly flowing PBS (approximately 5 mL/min). Blood removal was confirmed by the whitish color liver, heart and kidney of the observed mice. Following the perfusion, the brains were quickly removed, and the cerebellum was isolated from the brain on an ice plate. The cerebellar tissue excised was snap-frozen in liquid nitrogen for further AChE activity analysis [53]. On the day of AChE activity assessment, the cerebellar tissues of five groups (3–5 mice per group) were homogenized in the RIPA buffer (50-mM Tris HCl, pH 7.4, 140-mM NaCl, 1-mM EDTA, 0.5% Triton X-100 and 0.5% sodium deoxycholate) recommended by the manufacturer for extraction on ice, supplemented with inhibitors of protease and phosphatase activity. The tissues were sonicated with handheld tissue homogenizer and then centrifuged at 14,000 rpm for 30 min (4 °C) to get rid of tissue debris. The resulting supernatant was collected and used for AChE activity assessment [53,54]. Five groups of 3–5 mice/group were used for AChE activity estimations. For immunofluorescence, four animals from each group were further perfused and fixed with 4% paraformaldehyde solution, after being transcardially perfused with PBS. The brains were then postfixed in the same fixative (4% paraformaldehyde) for 48 h and subsequently exchanged with 10% sucrose solution for 3 consecutive days (4 °C). The brains were then stored at −80 °C for cryostat sectioning.

#### 2.4.2. Immunofluorescence Staining of Iba-1

A cryostat was used to slice the brains collected and stored earlier to 20-µm-thick coronal brain sections. The activation of microglia in the brain sections of the cerebellum was performed by immunofluorescence staining, analyzing Iba-1-positive microglia, as previously described methods [8,55]. Briefly, the brain sections were washed with PBS twice and incubated with a blocking solution 10% normal goat serum in PBS 0.3% Triton-X 100 at room temperature (RT). After 1 h, the blocking reagent was removed, and the sections were washed prior incubation with the primary antibody against Iba-1 (1:700) at 4 °C overnight. After incubation, the sections were washed with PBS twice and then incubated with corresponding fluorescent secondary antibody Alexa 488 anti-rabbit (1:1000) at RT for 1 h. Then the stained sections were subsequently washed and then mounted using Vectashield^®^ fluorescent mounting media. Consequently, fluorescent EVOS FL (Thermo Fisher Scientific) microscope was used to capture the images. Minimum of three cerebellar sections from each brain of total four animals/group were used to determine microglia activation. Activated microglia were determined in each section by randomly selecting three different fields of equal areas using the Image J software. In brief, circular outline was drawn around the area of interest, the mean green fluorescence emitted by Alexa Fluor^TM^ 488 was measured along with different adjacent background readings. The total corrected cellular fluorescence (TCCF) was then calculated using the following formula, TCCF = integrated density - (area of selected cell × mean fluorescence of the background readings) [55]. Results were represented as percentage fold increase from the control level.

### 2.5. Assessment of Cerebral Acetylcholinesterase (AChE) Activity in BTBR Mice

The commercially available acetylcholine assay kit was used, following the manufacturer’s instructions and as described previously [25]. The assay principle relates the AChE enzyme hydrolysis of ACh neurotransmitter to choline. Following the manufacturer’s instructions 5 μL of the supernatant of cerebellum tissue homogenate was added in to a 96-well plate. In each well, working reagent (45 μL) containing AChE assay buffer, and 50-µL reaction mix was added. Following 20–30 min incubation at 37 °C, VersaMax™ microplate reader (Molecular devices, San José, USA) was used to read the absorbance at two time points in a linear range (kinetic mode) to calculate the AChE activity in the sample. In the AChE activity assessment, 3–5 mice were used per group for four groups.

### 2.6. Statistics

For statistical comparisons, the software package SPSS 25.0 (IBM Middle East, Dubai, UAE) was used. For behavioral studies and AChE activity assessments, data were expressed as the mean value ± SEM. Normality analysis was carried out by assessing the sample distribution or skewness (−1.8 to +1.8 considered normally distributed). Following normality test, the effects of E100 were analyzed using two-way analysis of variance (ANOVA) with dose of drugs and animals (either BTBR or C57 mice) as the between-subjects factor, followed by post hoc analysis by Tukey’s test to calculate the statistical significance between various groups. In all tests, a *p*-value of less than 0.05 was considered statistically significant.

## 3. Results

### 3.1. Effects of E100 on Sociability and Social Novelty Impairments of BTBR Mouse in TCB

**Figure 1:**
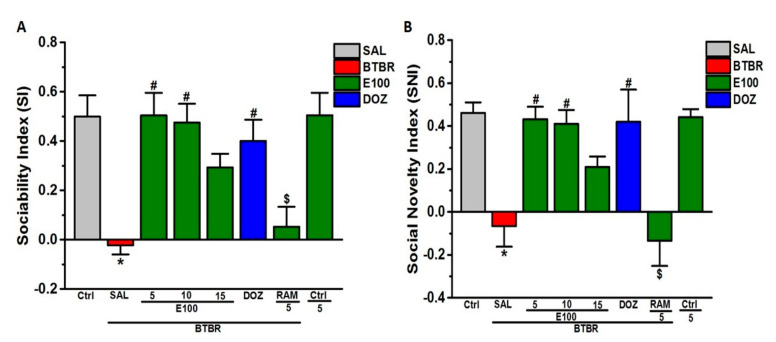
E100-improved sociability and social novelty deficits of BTBR mouse model of autism spectrum disorder (ASD). Mice were allowed to explore all 3 chambers for two cosecutive10 min sessions. The results attained were (**A**) Sociability index (SI) and (**B**) Social novelty index (SNI). C57BL/6 J (gray) received saline in Ctrl group, E100 (5,10 or 15 mg/kg, i.p. in green) or donepezil (DOZ) (1 mg/kg, i.p. in blue). All mice were treated for 21 days. Abrogative study of subchronic (21 days) systemic co-injection of RAM (10 mg/kg, i.p.) on the E100 (5 mg) provided improvement of (**A**) sociability and (**B**) social novelty. Values expressed as mean ± SEM (n = 5). ^*^ *p* < 0.05 compared to SI or SNI of saline-treated Ctrl mice. ^#^ *p* < 0.05 compared to SI or SNI of saline-treated BTBR mice. ^$^ *p* < 0.05 compared to E100 (5 mg)-treated BTBR mice.

Subchronic systemic injection effects of E100 (5, 10 and 15 mg/kg, i.p.) and DOZ (1 mg/kg, i.p.) on ASD-like sociability and social novelty impairments in the TCB task in BTBR mice are displayed in Figure 1A,B. Statistical analyses results revealed that subchronic pretreatment with E100 (5, 10 and 15 mg/kg, i.p.) and DOZ (1 mg/kg) prior to TCB increased SI significantly, with (*F*_(5,24)_ = 5.79; *p* < 0.001) (Figure 1A), enhancing sociability. Post hoc analysis showed that BTBR mice displayed significant sociability deficits expressed in form of SI value than SI values of control animals, with (*F*_(1,8)_ = 13.66; *p* < 0.05) (Figure 1A). However, E100 (5 and 10 mg/kg) and DOZ (1 mg/kg) significantly increased SI of BTBR mice when compared to saline treated BTBR mice group, with (*F*_(1,8)_ = 9.36; *p* < 0.05), (*F*_(1,8)_ = 8.74; *p* < 0.05) and (*F*_(1,8)_ = 6.21; *p* < 0.05), respectively (Figure 1A). Moreover, the results revealed that the enhancement in SI observed with E100 (5 mg/kg) was comparable to that shown with DOZ (1 mg/kg) and E100 (10 mg/kg), with (*F*_(1,8)_ = 0.46; *p* = 0.52) and (*F*_(1,8)_ = 0.26; *p* = 0.63), respectively (Figure 1A). Notably, E100 (15 mg/kg) failed to restore sociability deficits of BTBR mice, with (*F*_(1,8)_ = 4.21; *p* = 0.07) and than the E100 (5 mg)-treated BTBR mice (Figure 1A).

Interestingly, subchronic systemic co-administration with the brain penetrant H_3_R agonist RAM (10 mg/kg, i.p.) counteracted the E100 (5 mg)-provided sociability improvement, with (*F*_(1,8)_ = 10.90; *p <* 0.05) (Figure 1A). Similarly, the statistical analyses of the results exhibited that subchronic pretreatment with E100 (5, 10 and 15 mg/kg, i.p.) and DOZ (1 mg/kg) prior to TCB significantly enhanced social novelty by elevating SNI, with (*F*_(5,24)_ = 8.90; *p* < 0.001) (Figure 1B). Further post hoc analyses indicated that BTBR mice demonstrated significantly impaired social novelty preference when compared to the control mice, with (*F*_(1,8)_ = 6.69; *p* < 0.05) (Figure 1B). However, improvement in social novelty preference was achieved following subchronic systemic pretreatment of BTBR mice with E100 (5 and 10 mg/kg, i.p.) and DOZ (1 mg/kg, i.p.), with (*F*_(1,8)_ = 8.65; *p <* 0.05), (*F*_(1,8)_ = 5.47; *p <* 0.05) and (*F*_(1,8)_ = 5.37; *p <* 0.05), respectively (Figure 1B). E100 (15 mg/kg) failed to restore social novelty deficits of BTBR mice, with (*F*_(1,8)_ = 1.83; *p* = 0.21) (Figure 1B). As depicted in Figure 1B and in the post hoc analyses observation, the E100 (5 mg)-provided improvement of social novelty assessed by SNI was nullified by RAM (10 mg/kg, i.p.), with (*F*_(1,8)_ = 7.08; *p <* 0.05) as compared to the E100 (5 mg)-treated BTBR mice (Figure 1B). Interestingly, subchronic systemic pretreatment of control mice with the most promising dose of E100 (5 mg/kg, i.p.) did not alter SI or SNI than saline-treated control mice (*p* > 0.05).

### 3.2. Effects E100 on Stereotyped Repetitive and Obsessive-Compulsive Behaviors of BTBR Mouse in MBB and NST

**Figure 2:**
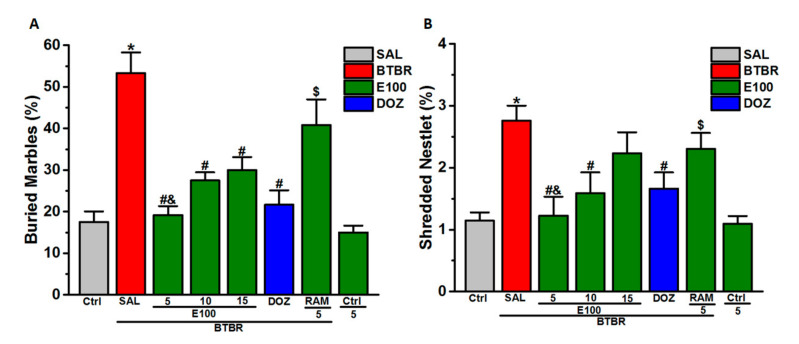
E100 mitigated stereotyped repetitive behavior in marble burying behavior (MBB) and attenuated increased obsessive-compulsive features in nestlet-shredding test (NST). (**A**) Repetitive marble-burying and (**B**) obsessive compulsive nestlet-shredding behavioral observations were evaluated after a 30-min testing session. BTBR mice (red) showed significant elevated stereotyped, repetitive and compulsive behaviors compared to Ctrl mice (gray). All mice were administered subchronically for 21 days with E100 (at a dose of 5, 10 or 15 mg/kg, i.p in green) or DOZ (1 mg/kg, i.p. in blue). Effects of subchronic (21 days) systemic co-injection of RAM (10 mg/kg, i.p.) on the E100 (5 mg)-provided decrease of stereotyped repetitive and compulsive behaviors of BTBR mice were displayed in (A) MBB and (B) NST. Values are expressed as mean ± SEM (n = 6). ^*^ *p* < 0.05 compared to saline-treated Ctrl mice. ^#^ *p* < 0.05 compared to saline-treated BTBR mice. ^&^ *p* < 0.05 compared to E100 (10 or 15 mg)-treated BTBR mice. ^$^ *p* < 0.05 compared to E100 (5 mg)-treated BTBR mice.

The subchronic systemic administration effects of E100 (5, 10 or 15 mg/kg, i.p.) or DOZ (1 mg/kg, i.p.) on the elevated repetitive behavior of BTBR mice in MBB is shown in Figure 2A. Statistical analyses results showed that subchronic pretreatment with 5, 10 or 15 mg/kg of E100 or 1 mg/kg DOZ prior to MBB significantly reduced the marbles buried increased percentage by BTBR mice, with (*F*_(5,24)_ = 14.22; *p* < 0.001) (Figure 2A). BTBR mice significantly buried more marbles than the control mice, with (*F*_(1,8)_ = 34.37; *p* < 0.05). However, pretreatment with E100 (5, 10 or 15 mg/kg, i.p.) and DOZ (1 mg/kg, i.p.) significantly obliterated the percentage of marbles increased buried by BTBR mice when compared to saline-treated BTBR mice, with (*F*_(1,8)_ = 29.41; *p* < 0.05), (*F*_(1,8)_ = 19.61; *p* < 0.05), (*F*_(1,10)_ = 13.24; *p* < 0.05) and (*F*_(1,8)_ = 22.85; *p* < 0.05), respectively (Figure 2A).

Moreover, the E100 (5 mg)-provided decrease in the percentage of buried marbles was comparable to the DOZ-provided effect, with (*F*_(1,8)_ = 0.12; *p* = 0.73) and was entirely abrogated by RAM, with (*F*_(1,8)_ = 2.08; *p* = 0.18) and as compared with saline-treated BTBR mice (Figure 2A). Furthermore, the effect observed with 5 mg/kg was significantly higher than that witnessed with 10 and 15 mg/kg, with (*F*_(1,8)_ = 6.75; *p* < 0.05) and (*F*_(1,8)_ = 7.76; *p* < 0.05), respectively (Figure 2A). Notably, subchronic systemic pretreatment of control mice with the best effective dose of E100 (5 mg/kg, i.p.) did not alter percentage of buried marbles in MBB as compared to saline-treated control mice (*p* > 0.05). Similarly, the subchronic systemic administration effect of E100 (5, 10 or 15 mg/kg, i.p.) or DOZ (1 mg/kg, i.p.) on the percentage escalation of shredded nestlet was evaluated in NST (Figure 2B). The statistical analyses results showed that subchronic pretreatment with E100 (5 and 10, /kg, i.p.) and DOZ (1 mg/kg) prior to NST significantly ameliorated compulsive like behavior by decreasing the percentage of cotton shredded (*F*_(5,24)_ = 4.26; *p* < 0.01) (Figure 2B). Post hoc analyses showed that BTBR mice significantly shredded more nestlet than the control mice, with (*F*_(1,8)_ = 27.94; *p* < 0.05). However, BTBR mice pretreated with E100 (5 or 10 mg/kg, i.p.) demonstrated lower percentage of shredded nestlet that was significant than BTBR mice treated with saline, with (*F*_(1,8)_ = 12.72; *p* < 0.05) and (*F*_(1,10)_ = 6.63; *p* < 0.05), respectively (Figure 2B). Moreover, the effect observed with 5 mg/kg was comparable to that witnessed with 10 mg/kg, with [*F*_(1,8)_ = 0.54; *p* = 0.48] and was significantly higher than that witnessed with 15 mg/kg, with (*F*_(1,8)_ = 6.11; *p* < 0.05) (Figure 2B). No significant difference was detected in the E100-provided effect on the nestlet shredded percentage between the two doses of 10 mg/kg and 15 mg/kg, with *p* = 0.17 (Figure 2B).

Moreover, subchronic systemic administration of DOZ (1 mg/kg, i.p.) attenuated the percentage of shredded nestlet significantly in BTBR mice, with (*F*_(1,8)_ = 7.79; *p* < 0.05). The post hoc analyses revealed that the subchronic systemic co-administration of RAM (10 mg/kg, i.p.), countered the decrease in nestlet shredded percentage provided by the E100 as shown in Figure 2B, with (*F*_(1,10)_ = 6.05; *p* < 0.05) as compared with E100 (5 mg)-treated BTBR mice (Figure 2B). Notably, subchronic systemic pretreatment of control mice with the most promising dose of E100 (5 mg/kg, i.p.) did not alter percentage of shredded nestlets in NST than saline-treated control mice (*p* > 0.05).

### 3.3. Effects of E100 on Anxiety Levels and Locomotor Activity of BTBR Mice in EPMT

**Figure 3:**
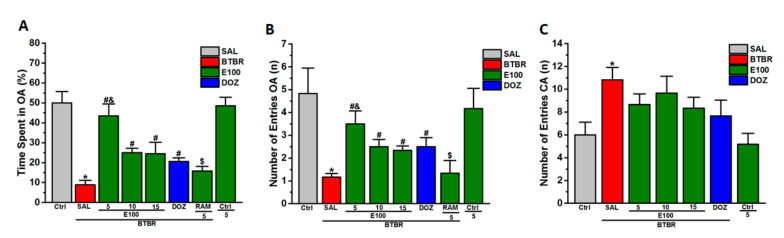
Effects of E100 and DOZ pretreatment on anxiety levels and locomotor activity in BTBR mice in EPMT test. BTBR mice (red) demonstrated significant elevated anxiety-related behavior and hyperactivity compared to Ctrl mice (gray). Subchronic (21 days) administration of E100 (5, 10 or 15 mg/kg, i.p. in green) or DOZ (1 mg/kg, i.p., in blue) attenuated (**A**) the decreased percentage of time spent exploring the open arms of the EPMT, (**B**) the decreased frequency of open arms entries and (**C**) failed to decrease the increased number of visits into the closed arms in BTBR mice. Effects of subchronic (21 days) systemic co-injection of RAM (10 mg/kg, i.p.) on the E100 (5 mg)-provided elevation of percentage of (**A**) time spent in open arms or (**B**) frequency of entries into open arms of BTBR mice were evaluated in EPMT. Data are expressed as the mean ± SEM (n = 6). ^*^ *p* < 0.05 compared to saline treated Ctrl mice. ^#^ *p* < 0.05 compared to saline treated BTBR mice. ^&^ *p* < 0.05 compared to E100 (10 or 15 mg)-treated BTBR mice. ^$^ *p* < 0.05 vs. E100 (5 mg)-treated BTBR mice.

Figure 3A–C shows the observed effects of E100 (5, 10 or 15 mg/kg, i.p.) subchronic systemic treatment on the anxiety parameters of BTBR mice in the EPMT, including, the percentage of time spent in open arms (Figure 3A), the frequency of open arms entries (Figure 3B) and locomotor activity expressed as the frequency of closed arms entries (Figure 3C). The results observed showed that BTBR mice spent significantly less time and lower number of entries in open arms when compared to control mice, with (*F*_(1,8)_ = 36.82; *p* < 0.05) and (*F*_(1,8)_ = 8.83; *p* < 0.05), respectively (Figure 3A,B). However, post hoc analyses subsequently revealed that E100 when administered at 5, 10 or 15 mg/kg i.p. significantly altered the time percentage of exploring the open arms of the maze during a 5 min test period compared to saline-treated BTBR mice, with (*F*_(1,8)_ = 23.99; *p* < 0.05), (*F*_(1,8)_ = 5.33; *p* < 0.05) and (*F*_(1,8)_ = 17.91; *p* < 0.05), respectively (Figure 3A). Moreover, post-hoc evaluation revealed that, compared with control mice, the BTBR mice displayed a lower number of entries into the open arms, with [*F*_(1,8)_ = 8.83; *p* < 0.05) (Figure 3B). However, during 5 min test session the administration of E100 5, 10 or 15 mg/kg i.p. significantly increased the number of visits to the open arms of the EPM as compared to saline-treated BTBR mice, with (*F*_(1,8)_ = 13.24; *p* < 0.05), (*F*_(1,8)_ = 12.31; *p* < 0.05) and (*F*_(1,8)_ = 18.85; *p* < 0.05), respectively (Figure 3B). Interestingly, significant attenuation in the time spent exploring the open arms was observed in BTBR mice pretreated with DOZ (1 mg) compared to saline-treated BTBR mice, with (*F*_(1,8)_ = 14.28; *p* < 0.05) (Figure 3A). Further analyses of data describing the frequency of visits into the open arms of the maze yielded for DOZ (1 mg/kg, i.p.) higher number of visits into open arms when compared to saline-treated BTBR mice, with (*F*_(1,8)_ = 8.42; *p* < 0.05) (Figure 3B). As shown in Figure 3A,B, the E100 (5 mg)-provided improvement in anxiety levels was abrogated by RAM (10 mg/kg, i.p.) with (*F*_(1,8)_ = 10.74; *p* < 0.05) and (*F*_(1,8)_ = 6.17; *p* < 0.05), for time spent exploring open arms and frequency of open arm entries, respectively and than E100 (5 mg)-treated BTBR mice. Notably, similar number of closed arm visits that was not significantly different was observed following E100 (5, 10 or 15 mg/kg) and DOZ (1 mg/kg, i.p.) subchronic injections than saline treated BTBR mice, with (*F*_(1,8)_ = 1.90; *p* = 0.20), (*F*_(1,8)_ = 0.34; *p* = 0.57), (*F*_(1,8)_ = 2.46; *p* = 0.15) and (*F*_(1,8)_ = 2.73; *p* = 0.13), respectively (Figure 3C). Interestingly, subchronic systemic pretreatment of control mice with the most promising dose of E100 (5 mg/kg, i.p.) did not alter any of the tested parameters in EPMT and than saline-treated control mice (all *p* > 0.05).

### 3.4. Effects of E100 on Anxiety-Related Behavior and Locomotor Activity Parameters of BTBR Mice in OFT

**Figure 4:**
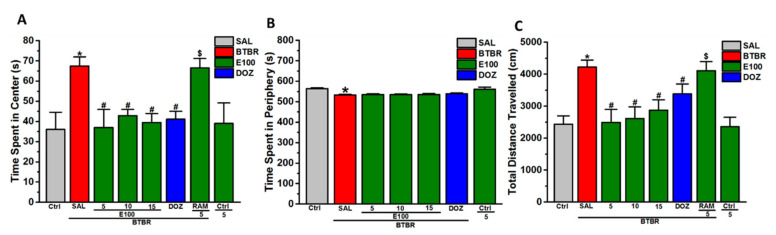
E100 and DOZ restored the abnormal anxiety and hyperactivity observed in BTBR mice in OFT. BTBR mice (red) demonstrated enhanced impulsive attitude and cognition deficits in as well as hyperactivity that was significant compared to Ctrl mice (gray). All mice were administered subchronically for 21 days with E100 (at a dose of 5, 10 or 15 mg/kg, i.p in green) or DOZ (1 mg/kg, i.p. in blue) and showed attenuation in the increased (**A**) time spent in the central zone as well as (**C**) the elevated total distance traveled, but failed to modulate the (**B**) time spent in the periphery in BTBR mice in the OFT. Effects of subchronic (21 days) systemic co-injection of RAM (10 mg/kg, i.p.) on the E100 (5 mg)-provided amelioration of time spent in the center and decreased (**C**) distance traveled of BTBR mice were evaluated in OFT. Data are expressed as the mean ±SEM (n = 4). ^*^ *p* < 0.05 compared to saline treated Ctrl mice. ^#^ *p* < 0.05 compared to saline treated BTBR mice. ^$^ *p* < 0.05 compared to E100 (5 mg)-treated BTBR mice.

In addition to EPMT, the OFT was used to test anxiety-associated behavior and locomotion. As seen in Figure 4A–C, no significant effects were observed in BTBR following the subchronic systemic exposure of to E100 (5, 10 and 15 mg/kg, i.p.) on time spent in the periphery (all *p* > 0.05) (Figure 4B). Contrary, analysis of variance revealed that pretreatment of BTBR with E100 (5, 10 and 15 mg/kg, i.p.) significantly spent lower percentage of time in the center of the arena, with (*F*_(1,6)_ = 6.96; *p* < 0.05), (*F*_(1,6)_ = 15.25; *p* < 0.05) and (*F*_(1,6)_ = 14.56; *p* < 0.05), respectively (Figure 4A). As shown in Figure 4A. Post hoc analyses revealed that subchronic systemic co-administration of RAM (10 mg/kg, i.p.) abrogated the E100 (5 mg)-provided decrease in the time spent in the center of arena, with (*F*_(1,6)_ = 6.42; *p* < 0.05) compared with E100 (5 mg)-treated BTBR mice (Figure 4A). Moreover, BTBR mice pretreated with E100 (5, 10 and 15 mg/kg, i.p.) and DOZ (1 mg/kg, i.p.) demonstrated significantly lower traveled distance when compared to saline-treated BTBR mice, with (*F*_(1,6)_ = 10.60; *p* < 0.05), (*F*_(1,6)_ = 10.57; *p* < 0.05), (*F*_(1,6)_ = 8.84; *p* < 0.05) and (*F*_(1,6)_ = 7.76; *p* < 0.05), respectively (Figure 4C). It is worth to mention that subchronic treatment of control mice with E100 (5 mg/kg, i.p.) did not significantly influence time spent in the center and periphery of the arena, neither the total distance moved than saline-pretreated control mice (all *p* > 0.05) (Figure 4A–C).

### 3.5. Effects of E100 on Cerebellar Acetylcholine Esterase Activity of BTBR Mice

**Figure 5:**
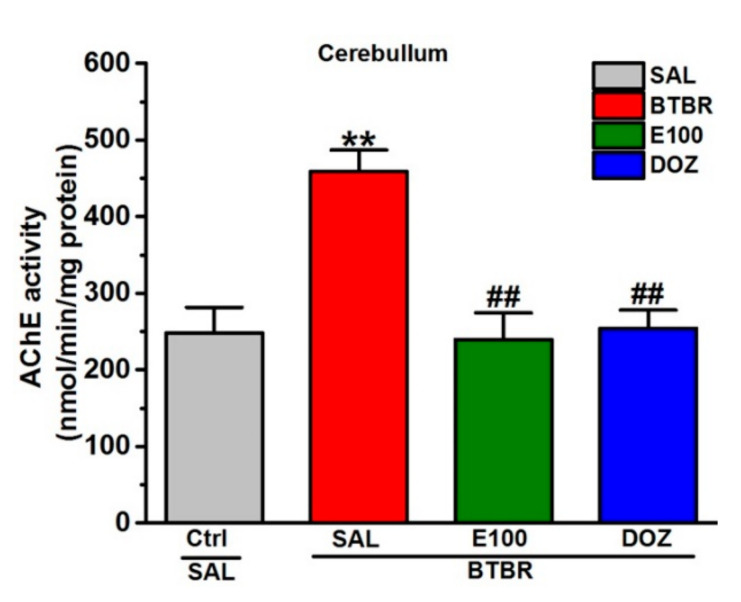
E100 reduced the enhanced acetylcholine esterase activity levels in BTBR mice cerebellum tissues. E100 (5 mg/kg, i.p.) provided inhibitory effects on AChE activity in the cerebellum of BTBR mice. Analysis revealed quantitively a significant increase (^**^ *p* < 0.01) in the AChE activity in cerebellum of BTBR mice compared to the control mice. E100 (5 mg/kg, i.p.) or DOZ (1 mg/kg, i.p.) subchronic (21 days) treatment significantly reduced (^##^ *p* < 0.01) this activity in BTBR mice compared to the saline-treated BTBR mice. Values are expressed as the percent mean ± SEM (n = 3–5).

Acetylcholine esterase activity in BTBR mice was assessed (Figure 5). The results demonstrated that BTBR mice significantly exhibited enhancement in the activity of acetylcholine esterase enzyme in cerebellar tissues of BTBR mice than control mice (*p* < 0.05) (Figure 5). However, E100 (5 mg/kg, i.p.) subchronic systemic treatment of BTBR mice significantly attenuated the acetylcholine esterase activity of BTBR mice than the saline-treated BTBR mice (*p* < 0.01) (Figure 5). Similarly, the AChE activity of BTBR mice was observed to be significantly decreased following subchronic systemic pretreatment with DOZ (1 mg/kg.) when compared with the saline-treated BTBR mice (*p* < 0.01) (Figure 5).

### 3.6. Effects of E100 on Cerebellar Activated Microglial Cells of BTBR Mice

**Figure 6:**
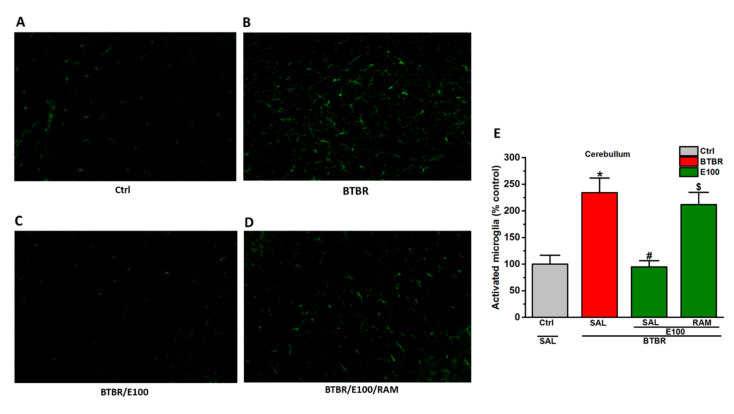
E100 decreased iba-1-positive microglial cells in cerebellum tissues of BTBR mice. (**A**) Immunofluorescent staining of cerebellar brain tissues with Iba-1 of BTBR mice; (**A**–**D**) Images show 20-μM-thick sections of cerebellum of different groups; (**E**) Densitometric assessment of fluorescent intensity was carried out to quantify activated microglia by Image J. (**B**,**E**) Significant increase (^*^ *p* < 0.05) in the number of activated microglia was observed in cerebellum of BTBR mice compared to Ctrl mice. (**C**,**E**) E100 (5 mg/kg, i.p.) subchronic treatment significantly reduced (^#^ *p* < 0.05) activated microglia in BTBR mice. (**D,E**) Co-injection of RAM (10 mg/kg, i.p.) for 21 days significantly reversed E100 (5 mg)-provided mitigation of activated microglia in BTBR mice as evidenced by the increased Iba-1-positive cells expression. Data expressed as the percent mean ± SEM (n = 3).

Microglial responses in BTBR mice was assessed (Figure 6B). Quantification of Iba-1 revealed that BTBR mice displayed a significant enhancement in the expression of iba-1-positive microglia, which is a marker of activated microglia (*p* < 0.05) (Figure 6B). Whereas E100 (5 mg/kg, i.p.) subchronic systemic treatment of BTBR mice ameliorated microglial activation in cerebellum than the saline-treated BTBR mice (*p* < 0.05) (Figure 6C). Moreover, subchronic systemic co-administration of RAM (10 mg/kg, i.p.) entirely reversed the effects of E100 (5 mg/kg, i.p.) on microglial activation than the E100 (5 mg)-treated BTBR animals (*p* < 0.05) (Figure 6D).

## 4. Discussion

Our results showed that E100 significantly improved social deficits exhibited by BTBR mice. This was evident by significant enhancement in sociability as well as social novelty behavior provided by dose of 5 mg/kg or 10 mg/kg. Importantly, the effect of E100 in the improvement of sociability and social novelty observed was not dose-dependent, since E100 (15 mg/kg, i.p) failed to further increase sociability or social novelty preference. Accordingly, the higher dose of E100 (15 mg/kg) may display off-target effects, such as the possibility of antimuscarinic effects, which could explain the decreased effects of E100 on all observed ASD-like features of tested BTBR mice following administration of higher doses. Therefore, future evaluation of antimuscarinic effect for E100 is essential to further elucidate the pharmacological properties of E100. In fact, E100 (5 mg/kg) was shown to be the optimal dose for providing enhancing effects on sociability as well as social novelty. The observed results regarding the doses are in agreement with previous results of a memory-enhancing effect observed for UW-MD-72, for which the effect of lower dose (1.25 mg/kg) was significantly higher when compared to the higher doses 2.5 and 5 mg/kg [41]. In a further experiment, the enhancement in sociability and social novelty provided by E100 (5 mg, being the optimal dose that alleviated the assessed behavioral deficits) were completely reversed when mice were co-administered with RAM, the brain-penetrant H_3_R agonist. The observed results support the effect of E100 to release several neurotransmitters, such as HA and ACh, provided by the inhibition of the H_3_R. These brain neurotransmitters have shown to be implicated in the regulation of arousal and cognitive processes in several previous studies. Earlier experimental study revealed that antagonism of H_3_R attenuated impaired social behaviors in rodents exposed to phencyclidine (PCP) [56], a finding that is in line with current results. Notably, the reference drug DOZ improved both social impairment parameters of BTBR mice following subchronic systemic treatment.

Recent studies revealed that cholinergic deficit and low levels of brain ACh are present in BTBR mice [57]. In addition to the well-established positive effect of AChEIs on cognition in wealth of studies, DOZ treatment was reported by Reidel et al. to relieve social memory deficiency [58]. This evidence fits with our findings, as E100 has AChE inhibitory property as DOZ, may explain the social deficits improvement observed in E100 (5 mg/kg, i.p.) pretreated BTBR mice. Moreover, the selective H_3_R antagonist JNJ-10181457 was reported in a previous study to normalize ACh neurotransmission and positively impact cognition [59], supporting our results of E100. These therapeutic effects of E100 may be explained by the modulation of histaminergic and cholinergic systems, hence reduced cognitive rigidity, that consequently increase social attention and elevate sociability levels.

Moreover, the cholinergic activity enhancement exerted by E100 in BTBR mice and the accompanied possible influence on cognitive impairment linked with sociability deficits was correlated to AChE activity evaluation in the cerebral tissues. The results demonstrated a significantly diminished AChE activity in E100 (5 mg)-treated mice when compared to BTBR mice and comparable to that observed for the reference drug DOZ. In view of the enzymatic role for degrading the neurotransmitter ACh, an earlier preclinical study reported that AChE inhibition amplified ACh levels in the BTBR mice synapse and therefore ameliorated the cognitive rigidity and social deficits [60].

Furthermore, E100 in BTBR mice also exhibited the same trend toward improvement in the repetitive and compulsive-like behaviors. Analysis of MBB and NST results revealed that E100 (5 mg/kg) exhibited the best behavioral improvement that was significant from effects of E100 (10 and 15 mg/kg). Therefore, all following abrogative studies applying co-administration of H_3_R agonist RAM were carried out with E100 at a dose of five milligram per kilogram as most promising optimal dose to avoid any off-target effects that may occur if higher doses (10 or 15 mg/kg) of E100 are used.

A previous study reported that acute administration of mAChR agonist oxotremorine attenuated the increased self-grooming and marble-burying behavior in BTBR mice. These findings propose that mAChR activation can alleviate certain repetitive behaviors [61]. This evidence may explain the current findings of E100 on repetitive behavior in MBB test, because E100 tend to facilitate the release of ACh through antagonism of H_3_ heteroreceptors and increase the level of ACh in synapse by exerting its inhibitory activity on AChE. The increased level of ACh may exert mAChR transduction showing the improvement in repetitive behavior observed. Additionally, and as mentioned earlier, several previous studies including our findings reported the positive effects of H_3_R antagonists on repetitive behaviors of valproic acid (VPA) mouse model of ASD in different mouse strains [8,24,25]. These findings suggest that the pharmacological manipulation of the histaminergic system by E100 due to its H_3_R antagonistic effect, may positively impact the elevated repetitive and compulsive-like behaviors in BTBR.

On the other hand, BTBR mice have been reported to have elevated oxidative stress with deficient enzymatic antioxidant response that is suggested to be associated with the exaggerated repetitive behavior [62]. Based on our previous finding of the capacity of E100 to modulate oxidative stress level in VPA exposed mice [25], the improvements observed in repetitive/compulsive behavior by E100 in BTBR is strongly supported.

BTBR mice display a variable profile of anxiety-like behaviors in comparison to C57 and other inbred mice strains. In the current study, BTBR mice spent less time and number of entries into open arms in EPMT, demonstrating high levels of anxiety. This observation was in agreement with previous studies [63,64]. Conversely, BTBR mice spent more time in the center of the arena of the open field, than C57 in OFT showing low levels of anxiety, reflecting impulsive behavior. This contradictory result is in line with several previous studies [65,66,67,68] and may be explained due to the stress condition exhibited by the EPMT over OFT. In fact, previous studies suggested that BTBR mice displayed high risk assessment behaviors (stretched postures and head-outs stretches), as response to high level of stress, reflecting exaggerated responses to stressors which may have influenced its responses in EPMT. Interestingly, E100 with all doses was able to restore these abnormal levels of anxiety in both tests, hence, our findings suggest interplay between HA and ACh level dysregulation with abnormal levels of anxiety and impulsive behavior observed in BTBR. This is supported by a recent report, in which ACh level decline in the prefrontal cortex region of BTBR mice exhibited attention deficit and impulsive behaviors [57]. In addition, DOZ and all doses of E100 failed to restore the locomotor hyperactivity displayed by BTBR mice in EPMT. However, the hyperactivity was totally restored in OFT with E100 (5,10 or 15 mg/kg, i.p.) or DOZ.

As mentioned earlier, both tests OFT and EPMT assessments are standard tools to analyze activity and anxiety profile in rodents. Open field determines behavior based on combined exploration and aversion against open and bright areas, whereas, in EPMT the behavior of rodents is based on openness combined with elevation predominantly related to anxiety [69]. This explains the sensitivity of each behavioral test in testing the effect of compounds on anxiety and psychomotor activity using same mice strain. Although E100 reduced the increased activity in BTBR mice, it failed to restore this hyperactivity in a previous preclinical experiment in C57 VPA-exposed mice in OFT [8] and may be explained with the variability in sensitivity of mouse strains to neuropsychiatric drugs. The latter finding agrees with a previous study in which the antidepressant-like effect of fluoxetine was compared in seven inbred mouse strains in the forced swimming test and the results revealed improvement in three strains only [70]. Interestingly, co-administration of the H_3_R agonist RAM reversed the elevated locomotor activity improvement exhibited by E100 (5 mg) in OFT, strongly correlating the regulation of both HA and ACh neurotransmitters with hyperactivity observed in BTBR mice.

Several studies now provide evidences of ongoing neuroinflammatory process in various brain regions involving microglial activation in individuals with ASD and several other neuropsychiatric diseases [71]. Accordingly, microglial activation can then result in a loss of connections or under-connectivity due to the sustained production of numerous mediators longer than usual, contributing to the loss of synaptic connections and subsequently neuronal cell death. This is important since numerous previous studies reported under-connectivity in individuals diagnosed with ASD [72]. Moreover, emerging evidence implicates compromised inter-hemispherical connectivity in some cases of ASD, where greater callosum reduction was proportional to severity of the disorder. Furthermore, histological analyses showed that the corpus callosum was absent in BTBR brains. Nevertheless, important finding suggested that genes regulating corpus callosum development specifically are not likely to be responsible for the abnormalities observed in the social and repetitive behaviors in BTBR, evidenced by postnatal surgical disconnection of the corpus callosal fiber tract in C57 mice that exhibited no social impairments or repetitive self-grooming that characterize BTBR mice [67]. These findings implicate other aspects of connectivity and brain development. Taken together, these exclusions support our findings that other CNS disruptions are involved in these behavioral abnormalities. Moreover, several previous studies indicated that cerebellar inflammation may alter social behavior in adult mice, as cerebellum is involved in executive and cognitive functions [8]. Importantly, previous studies revealed that numerous proinflammatory cytokines, including TNF-α, IL-1β, IL-6 and TGF-β, are abnormally elevated in the autistic brain [8,71]. Consistent with these findings and in a recent study, the dual-active AChEI and H_3_R antagonist E100 showed a significant modulation of levels assessed for these proinflammatory cytokines in mice, indicating that AChE as well as H_3_Rs are significantly contributing to the neuropsychiatric ASD-like features. Moreover, our current promising results strongly implicate the histaminergic and cholinergic systems dysregulation in the phenotype of ASD. Accordingly, one-way to control neuroinflammation is to reduce or inhibit microglial activation [8,16,24,25]. Therefore, it is plausible that by reducing brain inflammation and microglial activation, the neuro-destructive effects of chronic inflammation could be diminished and contribute for improved developmental outcomes. Consistent with previous studies that reported microglial activation in BTBR brains [73,74], our results demonstrated remarkably a higher expression Iba-1-positive microglial cells in BTBR cerebellum compared to C57, reflecting an increase in activated microglia. However, subchronic systemic administration of E100 (5 mg) significantly mitigated microglial activation in BTBR, illustrated by attenuation of the Iba-1 expression. In addition, co-administration of RAM reversed the suppression exhibited by E100 (5 mg). The latter data indicated clearly that E100 has the potential to inhibit microglial activation through antagonistic effect on H_3_Rs. The current findings complement the earlier observation of E100 (10 mg) on activated microglial cells of VPA-exposed C57 mice [8]. Since microglia constitutively express all four histamine receptors (H_1_R, H_2_R, H_3_R and H_4_R), the role of HA as neuron-to-glia alarm signal in the CNS has been well-established [75]. It has been reported in a recent study that HA counteracts proinflammatory microglia phenotype in the SOD1-G93A mouse model of Amyotrophic Lateral Sclerosis. They demonstrated that HA exhibits its beneficial action only in inflammatory SOD1-G93A microglia and in contrast elicits a proinflammatory effect in nontransgenic cells [76]. These findings comprehend an earlier study that demonstrated the dual role of HA under physiological and inflammatory context [77].

Additionally, HA deficiency in histidine decarboxylase knock out mice has been reported to make microglial cells more vulnerable to an inflammatory challenge, that further emphasizes the importance of HA in regulating microglial functions [78]. These reports fit well with our findings that implicate the involvement of brain HA and ACh in providing the neuroprotective action provided by E100 through its action to promote the release of brain HA and elevate the levels of ACh in synapse, as neuroprotection may be consequently responsible for the improvement in ASD-like behavioral outcomes in BTBR mice. In addition, the reported detection of α7-acetylcholine nicotinic receptors on microglia further supports our current significant results [79]. The initial observations show that a simultaneous interaction with the above two targets leads to symptomatic in vivo enhancements of behavioral autistic-like features in BTBR mice. However, the mitigating effects observed for the dual-acting compound E100 are not due to either its AChE-inhibiting or to its H_3_R-blocking properties alone, since it neither acts purely as an H_3_R antagonist, e.g., DL77 (non-imidazole-based H_3_R antagonist [24]) or ciproxifan (an imidazole-based H_3_R antagonist [23]) nor as an AChEI such as DOZ (used as a reference drug in the current study). Consequently, considering the different levels of brain neurotransmitters, including HA and ACh, in various brain areas of the BTBR mice with ASD-like behaviors as well as when following systemic administration with E100 would further contribute to explaining the neural intersections involved in the observed behavioral improvements. In addition, the proposed advantage of a dual-acting compound (e.g., E100) with combined affinities at the required targets over co-administration of two drugs are the straightforward single-compound pharmacokinetics. Thereby, supposed drug–drug interactions taking place with a combination therapy may be avoided.

## 5. Conclusions

The observed results in an idiopathic ASD mice model comprehend our previously obtained palliative effects of E100 in VPA-induced ASD in mice. Also, the current observations demonstrate that simultaneous targeting of the CNS histaminergic and cholinergic neurotransmissions is crucial for palliation of several ASD-like features, namely ASD-like social deficits and repetitive/compulsive behaviors and mitigated the levels of cerebellar microglial cells and AChE activity of tested BTBR mice used as idiopathic ASD model. Whether the alleviation of autistic-like behaviors in BTBR mice is obtained after administration of H_3_R antagonist or co-administration of an H_3_R antagonist and an AChEI was beyond the scope of this project and will require dose-finding experiments for several ratios of the combination of AChEIs and H_3_R antagonist. Further in vivo assessments on brain levels of ACh as well as HA in BTBR mice following different systemic treatments of test compound as well as reference drugs including a standard H_3_R antagonist (e.g., pitolisant) are still needed to evaluate whether multiple-active compounds, e.g., E100, is superior to AChEIs or H_3_R antagonists when administered alone.

## References

[1] Lord C., Cook E.H., Leventhal B.L., Amaral D.G. (2000). Autism spectrum disorders. Neuron.

[2] Nestler E.J., Hyman S.E. (2010). Animal models of neuropsychiatric disorders. Nat. Neurosci..

[3] Sheldrick R.C., Carter A. (2018). State-level trends in the prevalence of autism spectrum disorder (ASD) from 2000 to 2012: A reanalysis of findings from the autism and developmental disabilities network. J. Autism Dev. Disord..

[4] Xu G., Strathearn L., Liu B., Bao W. (2018). Prevalence of autism spectrum disorder among us children and adolescents, 2014–2016. JAMA.

[5] Eissa N., Al-Houqani M., Sadeq A., Ojha S.K., Sasse A., Sadek B. (2018). Current enlightenment about etiology and pharmacological treatment of autism spectrum disorder. Front. Neurosci..

[6] Amodeo D.A., Jones J.H., Sweeney J.A., E Ragozzino M. (2012). Differences in BTBR T+ tf/J and C57BL/6J mice on probabilistic reversal learning and stereotyped behaviors. Behav. Brain Res..

[7] McFarlane H.G., Kusek G.K., Yang M., Phoenix J.L., Bolivar V.J., Crawley J.N. (2008). Autism-like behavioral phenotypes in BTBR T+tf/J mice. Genes Brain Behav..

[8] Eissa N., Azimullah S., Jayaprakash P., Jayaraj R.L., Reiner D., Ojha S.K., Beiram R., Stark H., Łażewska R., Kieć-Kononowicz K. (2019). The dual-active histamine H3 receptor antagonist and acetylcholine esterase inhibitor E100 ameliorates stereotyped repetitive behavior and neuroinflammmation in sodium valproate induced autism in mice. Chem. Biol. Interact..

[9] Kemper T., Bauman M.L. (1998). Neuropathology of infantile autism. J. Neuropathol. Exp. Neurol..

[10] Friedman S.D., Shaw D.W.W., Artru A.A., Dawson G., Petropoulos H., Dager S.R. (2006). Gray and white matter brain chemistry in young children with autism. Arch. Gen. Psychiatry.

[11] Mukaetova-Ladinska E.B. (2017). Silent lives: Why do we fail community-dwelling people with dementia?. Age Ageing.

[12] Panula P., Chazot P.L., Cowart M., Gutzmer R., Leurs R., Liu W.L.S., Stark H., Thurmond R.L., Haas H.L. (2015). International union of basic and clinical pharmacology. XCVIII. Histamine receptors. Pharmacol. Rev..

[13] Panula P., Rinne J., Kuokkanen K., Eriksson K.S., Sallmen T., Kalimo H., Relja M. (1997). Neuronal histamine deficit in Alzheimer’s disease. Neuroscience.

[14] Sadek B., Stark H. (2016). Cherry-picked ligands at histamine receptor subtypes. Neuropharmacology.

[15] Sadek B., Saad A., Sadeq A., Jalal F., Stark H. (2016). Histamine H3 receptor as a potential target for cognitive symptoms in neuropsychiatric diseases. Behav. Brain Res..

[16] Arrang J.-M., Garbarg M., Schwartz J.-C. (1983). Auto-inhibition of brain histamine release mediated by a novel class (H3) of histamine receptor. Nature.

[17] Lovenberg T.W., Roland B.L., Wilson S.J., Jiang X., Pyati J., Huvar A., Jackson M.R., Erlander M.G. (1999). Cloning and functional expression of the human histamine H3 receptor. Mol. Pharmacol..

[18] Berlin M., Boyce C.W., Ruiz M.D.L. (2011). Histamine H3 receptor as a drug discovery target. J. Med. Chem..

[19] Parmentier R., Anaclet C., Guhennec C., Brousseau E., Bricout D., Giboulot T., Bozyczko-Coyne D., Spiegel K., Ohtsu H., Williams M. (2007). The brain H3-receptor as a novel therapeutic target for vigilance and sleep-wake disorders. Biochem. Pharmacol..

[20] Alachkar A., Khan N., Łażewska D., Kieć-Kononowicz K., Sadek B. (2019). Histamine H3 receptor antagonist E177 attenuates amnesia induced by dizocilpine without modulation of anxiety-like behaviors in rats. Neuropsychiatr. Dis. Treat..

[21] Alachkar A., Łażewska D., Kieć-Kononowicz K., Sadek B. (2017). The histamine H3 receptor antagonist E159 reverses memory deficits induced by dizocilpine in passive avoidance and novel object recognition paradigm in rats. Front. Pharmacol..

[22] Alachkar A., Azimullah S., Ojha S., Beiram R., Łażewska D., Kieć-Kononowicz K., Sadek B. (2019). The neuroprotective effects of histamine H3 receptor antagonist E177 on pilocarpine-induced status epilepticus in rats. Molecules.

[23] Baronio D., Castro K., Gonchoroski T., De Melo G.M., Nunes G.D.F., Bambini-Junior V., Gottfried C., Riesgo R. (2015). Effects of an H3R antagonist on the animal model of autism induced by prenatal exposure to valproic acid. PLoS ONE.

[24] Eissa N., Jayaprakash P., Azimullah S., Ojha S.K., Al-Houqani M., Jalal F.Y., Łażewska D., Kieć-Kononowicz K., Sadek B. (2018). The histamine H3R antagonist DL77 attenuates autistic behaviors in a prenatal valproic acid-induced mouse model of autism. Sci. Rep..

[25] Eissa N., Azimullah S., Jayaprakash P., Jayaraj R.L., Reiner D., Ojha S., Beiram R., Stark H., Łażewska D., Kieć-Kononowicz K. (2020). The dual-active histamine H3 receptor antagonist and acetylcholine esterase inhibitor e100 alleviates autistic-like behaviors and oxidative stress in valproic acid induced autism in mice. Int. J. Mol. Sci..

[26] Shah A., Wing L. (2006). Psychological approaches to chronic catatonia-like deterioration in autism spectrum disorders. Int. Rev. Neurobiol..

[27] Wang L., Almeida L.E.F., Spornick N.A., Kenyon N., Kamimura S., Khaibullina A., Nouraie M., Quezado Z. (2015). Modulation of social deficits and repetitive behaviors in a mouse model of autism: The role of the nicotinic cholinergic system. Psychopharmacology.

[28] Chen R., Davis L.K., Guter S., Wei Q., Jacob S., Potter M.H., Cox N.J., Cook E.H., Sutcliffe J.S., Li B. (2017). Leveraging blood serotonin as an endophenotype to identify de novo and rare variants involved in autism. Mol. Autism.

[29] Cavalli A., Bolognesi M.L., Minarini A., Rosini M., Tumiatti V., Recanatini M., Melchiorre C. (2008). Multi-target-directed ligands to combat neurodegenerative diseases. J. Med. Chem..

[30] A Ellenbroek B., Ghiabi B. (2015). Do Histamine receptor 3 antagonists have a place in the therapy for schizophrenia?. Curr. Pharm. Des..

[31] Koziol L.F., Budding D., Andreasen N., D’Arrigo S., Bulgheroni S., Imamizu H., Ito M., Manto M., Marvel C., Parker K. (2014). Consensus paper: The cerebellum’s role in movement and cognition. Cerebellum.

[32] Wang S.S.-H., Kloth A.D., Badura A. (2014). The cerebellum, sensitive periods, and autism. Neuron.

[33] Lucchina L., Depino A.M. (2014). Altered peripheral and central inflammatory responses in a mouse model of autism. Autism Res..

[34] Shi L., Smith S.E.P., Malkova N., Tse D., Su Y., Patterson P.H. (2009). Activation of the maternal immune system alters cerebellar development in the offspring. Brain Behav. Immun..

[35] Miller H.L., E Ragozzino M., Cook E.H., Sweeney J.A., Mosconi M.W. (2015). Cognitive set shifting deficits and their relationship to repetitive behaviors in autism spectrum disorder. J. Autism Dev. Disord..

[36] Gadad B.S., Hewitson L., Young K.A., German D.C. (2013). Neuropathology and animal models of autism: Genetic and environmental factors. Autism Res. Treat..

[37] Fernández M., Sierra-Arregui T., Peñagarikano O. (2019). The cerebellum and autism: More than motor control. Behavioral Neuroscience.

[38] Kuder K.J., Łażewska D., Latacz G., Schwed J.S., Karcz T., Stark H., Karolak-Wojciechowska J., Kieć-Kononowicz K. (2016). Chlorophenoxy aminoalkyl derivatives as histamine H3R ligands and antiseizure agents. Bioorganic Med. Chem..

[39] Łażewska D., Jończyk J., Bajda M., Szałaj N., Wieckowska A., Panek D., Moore C., Kuder K.J., Malawska B., Kieć-Kononowicz K. (2016). Cholinesterase inhibitory activity of chlorophenoxy derivatives—Histamine H3 receptor ligands. Bioorganic Med. Chem. Lett..

[40] Khan N., Saad A., Nurulain S.M., Darras F.H., Decker M., Sadek B. (2016). The dual-acting H3 receptor antagonist and AChE inhibitor UW-MD-71 dose-dependently enhances memory retrieval and reverses dizocilpine-induced memory impairment in rats. Behav. Brain Res..

[41] Sadek B., Khan N., Darras F.H., Pockes S., Decker M. (2016). The dual-acting AChE inhibitor and H3 receptor antagonist UW-MD-72 reverses amnesia induced by scopolamine or dizocilpine in passive avoidance paradigm in rats. Physiol. Behav..

[42] Sadek B., Bahi A., Schwed J.S., Walter M., Stark H. (2014). Anxiolytic and antidepressant-like activities of the novel and potent non-imidazole histamine H3 receptor antagonist ST-1283. Drug Des. Dev. Ther..

[43] Bahi A., Sadek B., Nurulain S.M., Łażewska D., Kieć-Kononowicz K. (2015). The novel non-imidazole histamine H3 receptor antagonist DL77 reduces voluntary alcohol intake and ethanol-induced conditioned place preference in mice. Physiol. Behav..

[44] Silverman J.L., Yang M., Lord C., Crawley J.N. (2010). Behavioural phenotyping assays for mouse models of autism. Nat. Rev. Neurosci..

[45] Thomas A., Burant A., Bui N., Graham D., Yuva-Paylor L.A., Paylor R. (2009). Marble burying reflects a repetitive and perseverative behavior more than novelty-induced anxiety. Psychopharmacology.

[46] Theoharides T.C., Tsilioni I., Patel A.B., Doyle R. (2016). Atopic diseases and inflammation of the brain in the pathogenesis of autism spectrum disorders. Transl. Psychiatry.

[47] Angoa-Pérez M., Kane M.J., Briggs D.I., Francescutti D.M., Kuhn D.M. (2013). Marble burying and nestlet shredding as tests of repetitive, compulsive-like behaviors in mice. J. Vis. Exp..

[48] Kim J.-W., Seung H., Kwon K.J., Ko M.J., Lee E.J., Oh H.A., Choi C.S., Kim K.C., Gonzales E.L., You J.S. (2014). Subchronic treatment of donepezil rescues impaired social, hyperactive, and stereotypic behavior in valproic acid-induced animal model of autism. PLoS ONE.

[49] Bahi A. (2013). Individual differences in elevated plus-maze exploration predicted higher ethanol consumption and preference in outbred mice. Pharmacol. Biochem. Behav..

[50] Bahi A. (2013). Increased anxiety, voluntary alcohol consumption and ethanol-induced place preference in mice following chronic psychosocial stress. Stress.

[51] Bahi A., Dreyer J.-L. (2012). Hippocampus-specific deletion of tissue plasminogen activator “tPA” in adult mice impairs depression- and anxiety-like behaviors. Eur. Neuropsychopharmacol..

[52] Bahi A., Dreyer J.-L. (2014). Chronic psychosocial stress causes delayed extinction and exacerbates reinstatement of ethanol-induced conditioned place preference in mice. Psychopharmacology.

[53] Haque M.E., Javed H., Azimullah S., Khair S.B.A., Ojha S. (2015). Neuroprotective potential of ferulic acid in the rotenone model of Parkinson’s disease. Drug Des. Dev. Ther..

[54] Javed H., Azimullah S., Khair S.B.A., Ojha S., Haque M.E. (2016). Neuroprotective effect of nerolidol against neuroinflammation and oxidative stress induced by rotenone. BMC Neurosci..

[55] A McCloy R., Rogers S., Caldon C.E., Lorca T., Castro A., Burgess A. (2014). Partial inhibition of Cdk1 in G2 phase overrides the SAC and decouples mitotic events. Cell Cycle.

[56] Griebel G., Pichat P., Pruniaux M.-P., Beeské S., Lopez-Grancha M., Genet E., Terranova J.-P., Castro A., Sánchez J.A., Black M. (2012). SAR110894, a potent histamine H3-receptor antagonist, displays procognitive effects in rodents. Pharmacol. Biochem. Behav..

[57] McTighe S.M., Neal S.J., Lin Q., Hughes Z.A., Smith D.G. (2013). The BTBR mouse model of autism spectrum disorders has learning and attentional impairments and alterations in acetylcholine and kynurenic acid in prefrontal cortex. PLoS ONE.

[58] Riedel G., Kang S., Choi D., Platt B. (2009). Scopolamine-induced deficits in social memory in mice: Reversal by donepezil. Behav. Brain Res..

[59] Galici R., Boggs J.D., Aluisio L., Fraser I.C., Bonaventure P., Lord B., Lovenberg T.W. (2009). JNJ-10181457, a selective non-imidazole histamine H3 receptor antagonist, normalizes acetylcholine neurotransmission and has efficacy in translational rat models of cognition. Neuropharmacology.

[60] Karvat G., Kimchi T. (2013). Acetylcholine elevation relieves cognitive rigidity and social deficiency in a mouse model of autism. Neuropsychopharmacology.

[61] Amodeo D.A., Yi J., Sweeney J.A., E Ragozzino M. (2014). Oxotremorine treatment reduces repetitive behaviors in BTBR T+ tf/J mice. Front. Synaptic Neurosci..

[62] Nadeem A., Ahmad S.F., Al-Harbi N.O., Attia S.M., Alshammari M.A., Al-Zahrani K.S., Bakheet S.A. (2019). Increased oxidative stress in the cerebellum and peripheral immune cells leads to exaggerated autism-like repetitive behavior due to deficiency of antioxidant response in BTBR T+ tf/J mice. Prog. Neuro-Psychopharmacol. Biol. Psychiatry.

[63] Benno R., Smirnova Y., Vera S., Liggett A., Schanz N. (2009). Exaggerated responses to stress in the BTBR T+ tf/J mouse: An unusual behavioral phenotype. Behav. Brain Res..

[64] Pobbe R.L., Defensor E.B., Pearson B.L., Bolivar V.J., Blanchard D.C., Blanchard R.J. (2011). General and social anxiety in the BTBR T+ tf/J mouse strain. Behav. Brain Res..

[65] Moy S.S., Nadler J.J., Young N.B., Pérez A., Holloway L.P., Barbaro R.P., Barbaro J.R., Wilson L.M., Threadgill D., Lauder J.M. (2007). Mouse behavioral tasks relevant to autism: Phenotypes of 10 inbred strains. Behav. Brain Res..

[66] Silverman J.L., Yang M., Turner S.M., Katz A.M., Bell D.B., I Koenig J., Crawley J.N. (2010). Low stress reactivity and neuroendocrine factors in the BTBR T+ tf/J mouse model of autism. Neuroscience.

[67] Yang M., Clarke A.M., Crawley J.N. (2009). Postnatal lesion evidence against a primary role for the corpus callosum in mouse sociability. Eur. J. Neurosci..

[68] Chadman K.K. (2011). Fluoxetine but not risperidone increases sociability in the BTBR mouse model of autism. Pharmacol. Biochem. Behav..

[69] Schmitt U., Hiemke C. (1998). Combination of open field and elevated plus-maze: A suitable test battery to assess strain as well as treatment differences in rat behavior. Prog. Neuro-Psychopharmacol. Biol. Psychiatry.

[70] Lucki I., Dalvi A., Mayorga A.J. (2001). Sensitivity to the effects of pharmacologically selective antidepressants in different strains of mice. Psychopharmacology.

[71] Eissa N., Sadeq A., Sasse A., Sadek B. (2020). Role of neuroinflammation in autism spectrum disorder and the emergence of brain histaminergic system. Lessons also for BPSD?. Front. Pharmacol..

[72] Rodriguez J.I., Kern J.K. (2011). Evidence of microglial activation in autism and its possible role in brain underconnectivity. Neuron Glia Biol..

[73] Zhang Y., Gao N., Kluetzman K., Mendoza A., Bolivar V.J., Reilly A., Jolly J.K., Lawrence D.A. (2013). The maternal autoimmune environment affects the social behavior of offspring. J. Neuroimmunol..

[74] Heo Y., Zhang Y., Gao D., Miller V.M., Lawrence D.A. (2011). Aberrant immune responses in a mouse with behavioral disorders. PLoS ONE.

[75] Hu W., Chen Z. (2017). The roles of histamine and its receptor ligands in central nervous system disorders: An update. Pharmacol. Ther..

[76] Apolloni S., Fabbrizio P., Amadio S., Napoli G., Verdile V., Morello G., Iemmolo R., Aronica E., Cavallaro S., Volonté C. (2017). Histamine regulates the inflammatory profile of SOD1-G93A microglia and the histaminergic system is dysregulated in amyotrophic lateral sclerosis. Front. Immunol..

[77] Barata-Antunes S., Cristóvão A.C., Pires J., Rocha S.M., Bernardino L. (2017). Dual role of histamine on microglia-induced neurodegeneration. Biochim. Biophys. Acta (BBA) Mol. Basis Dis..

[78] Frick L., Rapanelli M., Abbasi E., Ohtsu H., Pittenger C.M. (2016). Histamine regulation of microglia: Gene-environment interaction in the regulation of central nervous system inflammation. Brain Behav. Immun..

[79] Parada E., Egea J., Buendia I., Negredo P., Cunha A.C., Cardoso S., Soares M.P., López M.G. (2013). The microglial α7-acetylcholine nicotinic receptor is a key element in promoting neuroprotection by inducing heme oxygenase-1 via nuclear factor erythroid-2-related factor 2. Antioxid. Redox Signal..

